# The role of multimodal ultrasound in diagnosis of fetal bowel dilatation and prediction of adverse neonatal outcomes: A study of 86 cases in a series of 43,562 births

**DOI:** 10.1016/j.heliyon.2024.e27455

**Published:** 2024-03-03

**Authors:** Xuelei Li, Meng Zhou, Shanshan Wang, Chaoxue Zhang

**Affiliations:** a Department of Ultrasound, Anhui Province Maternity and Child Health Hospital, Anhui, China; b Department of Ultrasound, First Affiliated Hospital of Anhui Medical University, Anhui, China

**Keywords:** Fetal bowel dilatation, Multimodal ultrasound, 3D ultrasound volume imaging, Prenatal diagnosis, Prediction model

## Abstract

**Objective:**

To investigate the diagnostic utility of multimodal ultrasound for fetal bowel dilatation (FBD) in different parts of the bowel and to examine its prognostic potential in FBD.

**Methods:**

This retrospective study analyzed 86 fetuses with a dilated bowel identified via ultrasound in a 10-month postnatal follow-up. Both two- and three dimensional (2D and 3D, respectively) ultrasound volume imaging were used to characterize dilation across different bowel sections. The optimal intestinal diameter cut-off values for pathological bowel dilatation were determined and a predictive model for neonatal surgery was developed.

**Results:**

The 86 cases of dilatation were distributed as follows: duodenal (n = 36); jejunum/ileum (n = 35); and colonic (n = 15). Duodenal dilatations presented the earliest during pregnancy compared to the other 2 groups (24.4 versus [vs.] 29 vs. 33.7 weeks respectively; p < 0.05). Cases with small intestinal dilatation were delivered earlier than those with colonic dilatation (p < 0.05). Infants with duodenal dilatation had the lowest birth weight and the highest rate of multi-system abnormalities (30.6% vs. 5.7% vs. 20%; p < 0.001). More than one-half of the multi-system abnormalities had chromosomal abnormalities (multiple, 54% vs. single, 12.5%; p = 0.015). There were 2 stillbirths, 24 induced labors, 44 postnatal surgeries, and 18 normal cases after birth. In predicting adverse neonatal outcomes of jejunum/ileum dilatation using a cut-off value of 15.5 mm small intestine diameter, sensitivity was 81.5%, specificity was 62.5%, and the area under the receiver operating characteristic curve (AUC) was 0.762 (p < 0.05). For colonic dilatation, using a cut-off value of 21.5 mm colon diameter: sensitivity was 83.3%, specificity was 77.8%, and AUC was 0.861 (p < 0.05). In detecting jejunum/ileum and colonic obstruction, 3D ultrasound demonstrated significantly better diagnostic efficiency than 2D ultrasound (p < 0.05). Using the backward stepwise selection method, a predictive model for neonatal surgery in patients with jejunum/ileum and colonic dilatation was established: logit (P) = −1.58 + (2.32 × polyhydramnios) +(2.0 × ascites) +(1.14 × hyperechogenic bowel). The AUC for the prediction model was 0.874 (p < 0.05), with 76% sensitivity and 94.1% specificity.

**Conclusions:**

Duodenal dilatation occurred earlier, with a higher incidence of chromosomal abnormalities and multi-system abnormalities than dilatation of other parts of the bowel. 3D ultrasound played an important role in the detection of jejunum/ileum and colon obstructions. Clinical signs, including polyhydramnios, ascites, and strong echoes in the intestine, can be used to predict neonatal surgery.

## 1 Introduction

Fetal bowel dilatation (FBD) is typically detected on ultrasonography in the second or third trimester of pregnancy. It may arise from underlying pathological conditions and can serve as a sign of various fetal gastrointestinal disorders. FBD can indicate fetal intestinal obstruction; however, its clinical significance remains unclear. It may persist until delivery without leading to postnatal abnormalities or result in adverse neonatal outcomes, thereby complicating prenatal management and consultation [1,2]. Moreover, abnormalities in different parts of the digestive system often present with overlapping ultrasound features, further increasing the difficulty of prenatal diagnosis [2]. For example, fetal intestinal volvulus, a rare but acute condition, often leads to FBD with a nonspecific presentation, which may result in an incorrect or missed diagnosis. An incorrect or delayed diagnosis and inadequate intervention can increase the risk for neonatal mortality. Furthermore, the choice of delivery method and timing of surgery vary depending on the type of intestinal obstruction. Therefore, accurate identification of pathological intestinal dilatation is crucial. Unfortunately, comprehensive data regarding the prognosis of fetuses with complicated bowel dilatation are lacking. Promising prognostic factors remain elusive because some potential indicators of FBD have not yet been validated as useful predictors of neonatal outcomes owing to small sample sizes [3,4]. However, significant advances have been made in this area. Three-dimensional (3D) ultrasound provides better visualization of fetal abnormalities, and its inversion mode enables visualization of fluid-filled organs [5], which may improve the diagnostic efficacy of FBD. The present study used both two-dimensional (2D) and 3D ultrasound methods to diagnose and differentiate FBD. We aimed to evaluate the diagnostic efficacy of different ultrasound methods and signs associated with FBD, as well as to determine the optimal cut-offs for intestinal diameter and their predictive value for adverse neonatal outcomes. We also analyzed the neonatal outcomes and postoperative results for FBD at various anatomical locations (including the duodenum, jejunum/ileum, and colon), and developed a predictive model for neonatal surgery.

## 2 Methods

### 2.1 Study design and patient selection

**Fig. 1:**
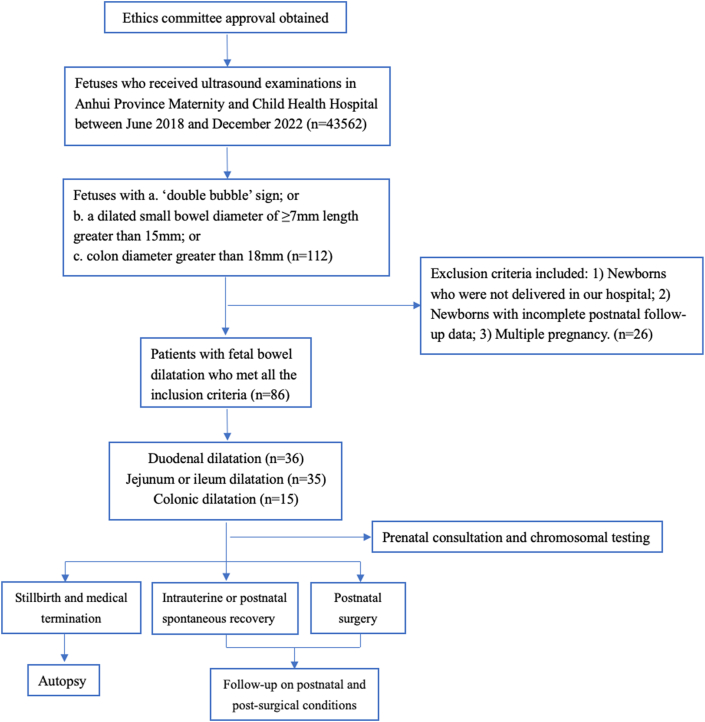
Flowchart of selection process of included patients.

This retrospective study analyzed fetuses and infants with FBD who underwent ultrasonographic examinations at the Anhui Province Maternity and Child Health Hospital (Anhui, China) between June 2018 and December 2022. Exclusion criteria were as follows: newborns who were not delivered at the authors' hospital; newborns with incomplete postnatal follow-up data; and multiple pregnancies. Among the 43,562 fetuses that underwent ultrasound examinations at the authors’ hospital, data from 86 with FBD were identified and included in this study (Fig. 1). This study was reviewed and approved by the Ethics Committee of the Anhui Province Maternity and Child Health Hospital (Approval number: YYLL2021-03-01-02). Informed consent was obtained from all patients for participation in the study and publication of anonymized case details and images. For participants under the age of consent (newborn infants), written informed consent was obtained from their parents or legal guardians.

### 2.2 Measurement and outcome

#### 2.2.1 Ultrasound system

Ultrasound examinations were performed using a Doppler ultrasound device (WS80A Elite and Samsung W10 color Doppler ultrasound, Samsung Healthcare, Seoul, Korea), equipped with a CV1-8A volume probe and C2-9 and C3-10 convex array probes. The device features “Crystal” and “Reality” Vue rendering modes. The “Crystal Vue” mode submenu includes 2 post-processing methods: grayscale and inversion.

#### 2.2.2 Evaluation of prenatal FBD

Based on disease location, dilatation was categorized into 3 groups: duodenal; jejunum/ileum; and colonic. Diagnostic criteria were as follows: 1) Duodenal dilatation: presence of the “double bubble sign” in the upper abdomen [6]. 2) Jejunum/ileum dilatation: a dilated small bowel with a diameter of ≥7 mm and length greater than 15 mm during the mid to late stages of pregnancy [7]. 3) Colonic dilatation: colon diameter greater than 18 mm in late pregnancy [8]. 4) Polyhydramnios: an amniotic fluid pocket depth >8 cm or an amniotic fluid index >25 cm. 5) Increased echogenicity of the intestine: the echo within the intestinal tube stronger than that of the bones [9]. 6) Fluid-filled level sign: local deposition of intestinal contents at the bottom of the intestinal lumen, with the fluid positioned above, forms a typical layered structure. 2D ultrasound was used to measure and evaluate fetal biological indicators.

Peak velocity of systolic blood flow in the middle cerebral artery (MCA-PSV) > 1.5MoM and umbilical artery S/D > 95th percentile were considered to be abnormal for intrauterine blood flow. When FBD was detected, echo and peristalsis of the intestinal contents were dynamically observed. The intestinal wall was magnified to assess layer clarity. Color Doppler flow imaging was used to visualize the course and spatial relationship of the superior mesenteric vein and artery, and to evaluate abdominal fluid and the echo intensity of the intestinal contents. When a double bubble sign appeared in the upper abdomen, the probe was adjusted to observe its connection with the pylorus. For 3D volume imaging of the transverse section of the dilated intestine, the surface mode was selected at a scanning angle of 60° to ensure high-quality scanning. The frame was magnified (encompassing the entire abdominal skin) for automatic scanning. To minimize spine shadowing, the fetal spine was maintained between the 3 and 9 o'clock positions, and the initial section of the dilated area was chosen as the acquisition plane. The acquired volume data were displayed in four images: A plane (the starting section of the sampled 2D plane); B plane (perpendicular to the A plane, showing the thickness within the volume data); C plane (perpendicular to both A and B planes); and 3D rendering. The 3D images were adjusted using a combination of the “Crystal Vue” and “Reality Vue” imaging modes. In the grayscale mode, the sampling line was placed inside the dilated intestine to observe the bowel interior and intestinal wall, whereas in the inversion mode, the sampling line was positioned on the exterior (closely attached) of the dilated intestine to observe external morphological changes.

#### 2.2.3 Prenatal and postnatal follow-up

Ultrasound examinations were performed biweekly after the discovery of FBD. In suspected cases of midgut volvulus, ultrasonographic examinations were performed weekly after 28 weeks’ gestation. Parents of fetuses with FBD were advised to consult a prenatal diagnosis clinic and undergo relevant genetic testing. The prenatal diagnosis was then compared with postnatal surgical outcomes or autopsy results following pregnancy termination and imaging findings. In newborns, parameters such as birth weight, gestational age at birth, digestive system complications, growth curves, and feeding patterns were monitored for up to 10 months. Adverse neonatal outcomes included medically advised pregnancy termination, stillbirth, neonatal death, chromosomal abnormalities, and postnatal surgery. Pregnancy terminations initiated solely on family request without a medical indication were not included in the subsequent analysis of adverse neonatal outcomes (receiver operating characteristic [ROC] curve analysis). Abnormal ultrasonographic features included strong echoes within the intestine, abnormal intestinal wall echoes, changes in intestinal peristalsis, fluid-filled level signs, and ascites. Cases were categorized as either isolated or complex based on the presence of abnormalities outside the digestive system.

### 2.3 Statistical analysis

Continuous variables are expressed as mean with standard deviation ($\bar{x}\pm s$) and compared using the *t*-test or Mann–Whitney *U* test. Categorical variables are expressed as frequency with proportion and were compared using the chi-squared test or Fisher's exact test. The areas under the ROC curve (AUC) for the pathological jejunum/ileum and colonic dilatation bowel diameters were analyzed and compared using the DeLong method. The optimal cut-off values for bowel diameter were determined using Youden's J index. Sensitivity and specificity were calculated to assess diagnostic performance. Logistic regression was performed and a backward stepwise selection method was used to obtain a prediction model for neonatal surgery. Differences with P < 0.05 were considered to be statistically significant. All statistical analyses were performed using SPSS (IBM Corporation, Armonk, NY, USA).

## 3 Results

### 3.1 Patients’ basic characteristics

Among the 86 fetuses with FBD, 36 (41.9%) presented with duodenal dilatation, 35 (40.7%) with jejunum/ileum dilatation, and 15 (17.4%) with colonic dilatation. Regarding the outcomes of these pregnancies, 2 were stillbirths (2.3%), 24 involved induced labor (27.9%), and 18 were either normal later in pregnancy or found to be normal postnatally (20.9%). Of the 2 stillbirths and 24 cases of induced labor, all diagnoses were confirmed via autopsy. Chromosome testing was performed in 42 cases (35 duodenal, 6 jejunal/ileal, and 1 colonic), revealing nine abnormalities in 9: trisomy 21 (n = 7); trisomy 18 (n = 1); and *ADSL* gene variation (n = 1). Notably, all 9 cases with chromosomal abnormalities were observed in the duodenal dilatation group. Furthermore, approximately one-half of the patients (44/86) required postnatal surgery.

The basic characteristics of each group are summarized in Table 1. Duodenal dilatation was detected earlier in pregnancy than in the other 2 groups (24.4 weeks versus [vs.] 29 weeks vs. 33.7 weeks, respectively; p < 0.001). Patients with small intestinal dilatation were delivered earlier than those with colonic dilatation; however, no significant difference was observed between the duodenal and jejunum/ileum dilatation groups. Duodenal dilatation cases had the lowest average birthweight (2433 ± 477 g; p = 0.007), the highest rate of complex FBD cases (30.6%, p < 0.001), while jejunum/ileum dilatation cases exhibited the highest rate of polyhydramnios (45.71%, p = 0.029). The mean bowel diameter was 19.34 ± 9.46 mm for jejunum/ileum dilatation and 20.73 ± 2.82 mm for colonic dilatation.

**Table 1:** Patients' basic characteristics.

	Duodenal dilatation (n = 36)	Jejunum/ileum dilatation (n = 35)	Colonic dilatation (n = 15)	P Value
Age of mother (years)	28 ± 5	29 ± 5	30 ± 4	0.744
Gestational age at diagnosis (weeks)	24.4 ± 2.4	29 ± 4.5	33.7 ± 4.9	＜0.001
Intestinal diameter at diagnosis (mm)	/	19.34 ± 9.46	20.73 ± 2.81	
Gestational age at birth (weeks)	35.7 ± 2.5	35.8 ± 2.6	38.7 ± 1.7	0.002
Birth weight (g)	2433 ± 477	2917 ± 469	2910 ± 786	0.007
Combination of other abnormalities				
Complex FBD	11 (30.6%)	2 (5.7%)	3 (20%)	＜0.001
Isolated FBD	25 (69.4%)	33 (94.3%)	12 (80%)	
Polyhydramnios	8 (22.22%)	16 (45.71%)	2 (13.33%)	0.029

### 3.2 Ultrasound signs and outcomes of duodenal dilatation

**Fig. 2:**
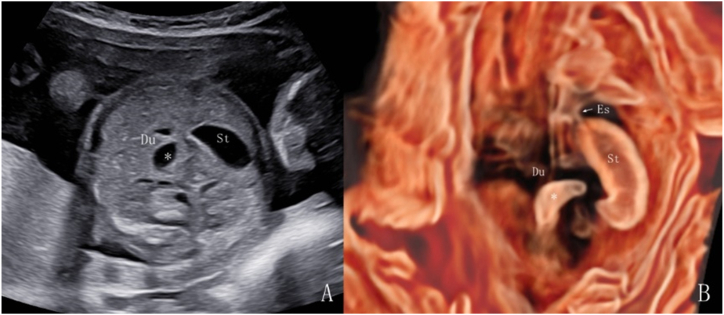
Fetus at 22 weeks and 2 days of gestation with proximal duodenal dilation. Postnatal surgery confirmed proximal narrowing of the descending part of the duodenum. A. 2D ultrasound shows the “double-bubble sign” in the upper abdomen. B. In 3D inversion mode, a clear connection is seen between the esophagus and the gastric bubble, and the proximal duodenal dilation appears as a “crescent moon” shape.

**Fig. 3:**
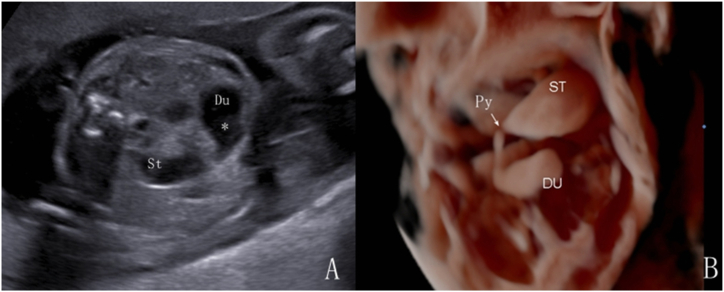
Fetus at 24 weeks of gestation with distal duodenal obstruction. Postnatal surgery confirmed horizontal duodenal atresia. A. 2D ultrasound shows the “double-bubble sign” in the upper abdomen. B. 3D volume imaging demonstrates the connection between the gastric bubble and the duodenum through the pylorus, with proximal dilation of the descending and horizontal parts of the duodenum forming a “C” shape.

A double bubble sign was observed on 2D ultrasonography in all 36 patients with duodenal dilatation (Fig. 2, Fig. 3A). In the 3D volume imaging inversion mode, the entire expanded duodenum can be visualized. The proximal duodenal obstruction exhibited a “crescent moon” shape change (Fig. 2B), while the distal obstruction exhibited a “C” shape change (Fig. 3B).

Among these cases, 11 were complex and presented additional abnormalities, as reported in Table 2. More than one-half of these complex cases also had chromosomal abnormalities (Table 3), with a significantly higher incidence than isolated cases (54.5% vs. 12.5%; p = 0.015). Regarding duodenal dilatation outcomes, 11 patients with complex intestinal dilatation underwent pregnancy termination. Additionally, medical induction was performed in 4 cases of isolated intestinal dilatation at the request of the families. One case showed normal intestinal restoration upon a two-week re-examination after the initial detection (without undergoing chromosomal testing and thus was not included in Table 3). One stillbirth occurred at 26 weeks’ gestation in a fetus with trisomy 21. Postnatally, 1 newborn experienced spontaneous recovery 2 weeks after birth, while 19 required surgical intervention, as detailed in Table 4. Thirteen of these cases were preterm births. Notably, 1 patient with duodenal atresia and abnormal intrauterine blood flow underwent emergency surgery at 35 weeks. The patient subsequently experienced cerebral hemorrhage and brain injury following surgery.

**Table 2:** Details of complex duodenal dilatation.

Combination of other abnormalities	n = 11
Cystic hygroma	1 (9.1%)
Heart anomalies	5 (45.4%)
Abnormal intrauterine blood flow^a^	2 (18.2%)
Abnormal femur and humerus^b^	1 (9.1%)
Incomplete ossification of the nasal bone	2 (18.2%)

a Peak velocity of systolic blood flow in the middle cerebral artery >1.5MoM and umbilical artery S/D > 95th percentile.

b Femur and humerus < −2 standard deviations of normal range at same gestational age.

**Table 3:** Incidence of chromosome abnormalities in isolated and complex duodenal dilatation (n = 35^a^).

	Isolated duodenal dilatation (n = 24)	Complex duodenal dilatation (n = 11)	P vale
Abnormal chromosome	3 (12.5%)	6 (54.5%)	0.015
Normal chromosome	21 (87.5%)	5 (45.5%)	

a Of the 36 duodenal dilatation cases, one was excluded due to normal intestinal restoration on the two-week follow-up after the initial detection, without chromosomal testing.

**Table 4:** Surgical diagnosis of Duodenum Dilatation Cases.

Surgical diagnosis	n = 19
Annular pancreas	2 (10.5%)
Duodenal diaphragm	6 (31.6%)
Duodenal atresia or stricture	10 (52.6%)
Malrotation	1 (5.3%)

### 3.3 Ultrasound signs and outcomes of jejunum/ileum dilatation

Among the 35 cases of jejunum/ileum dilatation, 25 (71.4%) fetuses presented with ≥1 type(s) of associated gastrointestinal abnormalities as detected on 2D ultrasound. These abnormalities included punctate echogenicity of the intestinal contents, ascites, changes in bowel movements (either disappearance or increase), echogenic changes in the bowel wall (segmental echogenicity enhancement or reduction), and fluid-filled level signs. Additionally, there was 1 case with a single umbilical artery and 1 case of hemivertebra.

**Fig. 4:**
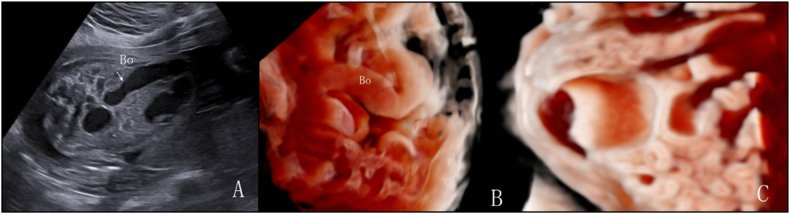
Fetus at 28 weeks and 5 days of gestation with small intestinal dilatation. Postnatal surgery confirmed distal jejunal atresia. A. 2D ultrasound shows dilated intestine with clear acoustic transmission through the intestinal contents. B. 3D imaging inversion mode demonstrates changes in the morphology of the dilated intestine. C. 3D imaging grayscale mode confirms the dilated intestinal segment as the small intestine with the smooth inner wall and the absence of haustra of colon.

**Fig. 5:**
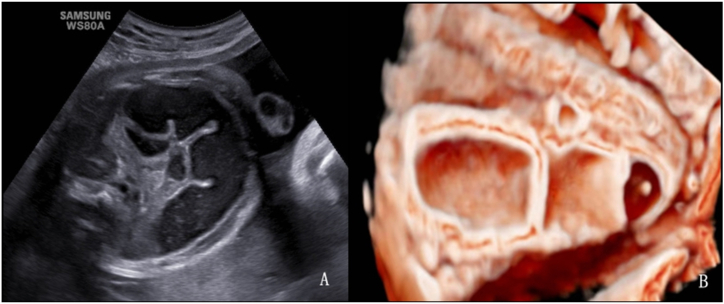
Fetus at 27 weeks and 3 days of gestation with small intestinal dilatation. Postnatal surgery confirmed ileal atresia. A. 2D ultrasound shows intestinal dilatation, with localized distribution of small bright spots within the intestinal lumen. During peristalsis, the walls of two adjacent intestines come into contact and protrude into the intestinal lumen. B. In 3D volume imaging in grayscale mode, the dilated intestinal segment is identified as the small intestine with the smooth inner wall.

**Fig. 6:**
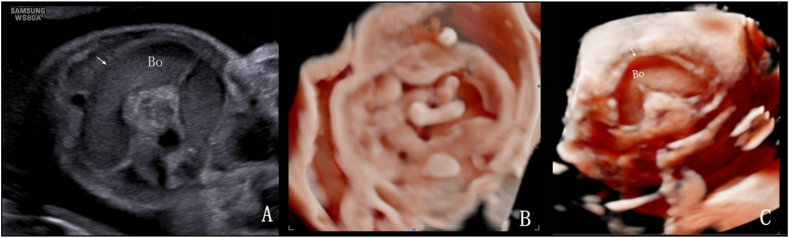
Fetus at 29 weeks and 4 days of gestation with small intestinal dilatation. Postnatal surgery confirmed terminal ileum torsion necrosis and mesenteric dysplasia. A. 2D ultrasound shows dilated intestine with the intestinal contents appearing densely packed with tiny light spots. B. In 3D imaging inversion mode, the dilated intestinal segment shows a “spiral sign”. C. 3D imaging in grayscale mode shows a smooth inner wall in the dilated intestine, indicating it is the small intestine with single-layer changes in the intestinal wall.

In 35 cases, 3D volume imaging in the grayscale mode revealed a smooth intestinal wall without haustra of the colon, whereas the inversion mode highlighted morphological changes in the dilated intestine. Fig. 4, Fig. 5 show two cases of small intestinal dilatation: the 2D ultrasound images (Fig. 4, Fig. 5A) provide foundational visualization of the intestinal dilatation, while 3D volume imaging in grayscale mode (Fig. 4, Fig. 5B) and in inversion mode (Fig. 4B) display distinctive characteristics of the intestinal wall. Of these patients, 23 were diagnosed with volvulus using ultrasonography. On 2D ultrasound, the “whirlpool sign” was observed in 10 cases, but only 6 exhibited the typical pattern of the superior mesenteric vein around the artery. The whirlpool sign disappeared during follow-up in 5 cases: 3 resolved and 2 developed into the “coffee bean sign”. Overall, 8 patients presented with the coffee bean sign, and 4 exhibited the whirlpool sign. In 3D inversion mode, 20 volvulus cases exhibited the “spiral sign”. In the 3D grayscale mode, 9 cases exhibited the disappearance or disarray of intestinal wall echoes and absent peristalsis, with bowel necrosis confirmed postnatally. Among these, 7 had MCA-PSV >1.5 MoM and were diagnosed with anemia after birth. Fig. 6 presents a case of small intestinal dilatation at 29 weeks and 4 days of gestation, subsequently confirmed by postnatal surgery as terminal ileum torsion necrosis and mesenteric dysplasia: a 2D ultrasound reveals a dilated intestine with densely packed tiny light spots (Fig. 6A), 3D inversion mode uncovers a “spiral sign” (Fig. 6B), and 3D grayscale mode shows single-layer changes in the intestinal wall (Fig. 6C). The 3 patients that resolved underwent upper gastrointestinal series post-birth and were diagnosed with malrotation. The incidence of the 3 ultrasound signs in patients with volvulus is summarized in Table 5, with the spiral sign having the highest detection rate (86.9%), suggesting greater sensitivity in identifying volvuli.

**Table 5:** Incidence of different ultrasound signs in patients with volvulus.

Ultrasound signs	n = 23	P value
Whirlpool sign	10 (43.5%)	P＜0.05
Coffee bean sign	8 (34.8%)	
Spiral sign	20 (86.9%)	

**Fig. 7:**
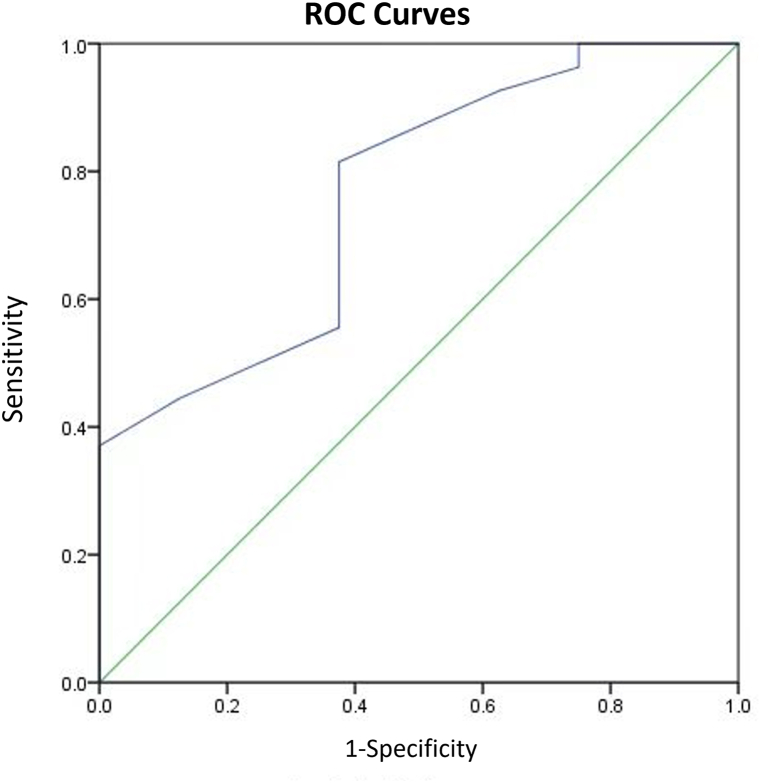
ROC curve of dilated small intestine diameter in predicting adverse pregnancy outcome in fetuses with jejunal or ileal dilatation.

Among patients with jejunum/ileum dilatation, 3 underwent medical termination of pregnancy at the request of the families, and 1 stillbirth occurred at 32 weeks' gestation. Eight cases resolved spontaneously between 24 and 33.5 weeks' gestation and the patients exhibited no symptoms postnatally. Twenty-three newborns required postnatal surgery: 4 cases of jejunal atresia (1 with volvulus); 5 cases of ileal atresia (2 with volvulus); and 14 cases of volvulus with necrosis in the twisted intestinal segment (6 with short or narrow mesentery, 5 with malrotation, and 3 idiopathic). Two infants with volvuli died postoperatively. Additionally, 5 surviving infants developed short bowel syndrome postoperatively. A ROC curve was generated using small intestine diameter to predict adverse neonatal outcomes (Fig. 7), yielding an AUC of 0.762 (95% confidence interval [CI] 0.574–0.949; p < 0.05). With an optimal cut-off value of 15.5 mm, the sensitivity and specificity were 81.5% and 62.5%, respectively. Cases that underwent pregnancy termination solely on families’ request were excluded from the ROC analysis.

### 3.4 Ultrasound signs and outcomes of colonic dilatation

**Fig. 8:**
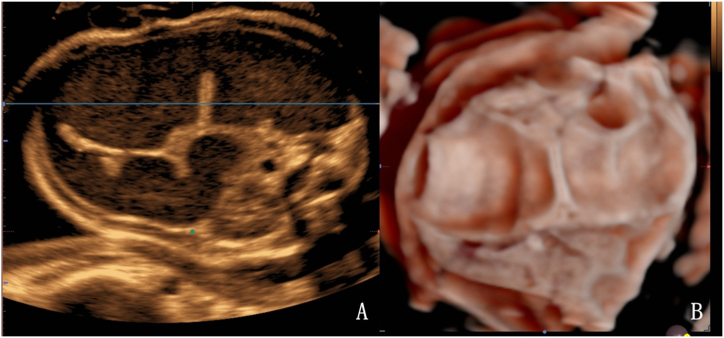
Fetus at 32 weeks of gestation with colonic dilatation. Postnatal surgery confirmed distal atresia of ascending colon. A. 2D ultrasound shows dilated intestine with dense, small bright spots within the intestinal lumen. B. In the grayscale mode of 3D ultrasound, the presence of haustra on the inner wall of the intestine is well displayed.

In the present study, most cases of colonic dilatation were detected after 29 weeks’ gestation. An exception was 1 case associated with VACTERL (including anal atresia, spina bifida, foot inversion, and pulmonary atresia with a ventricular septal defect) that was identified at 24 weeks. On 2D ultrasound, echogenic foci within the colon were observed in 9 cases, while 6 cases exhibited good acoustic transmission characterized by only fine speckles filling the colon (Fig. 8A). In the 3D grayscale mode, the haustra of the colon were visible in all cases (Fig. 8B). Notably, 5 cases initially misdiagnosed as small intestinal dilatation on 2D ultrasound were correctly identified as colonic dilatation after 3D ultrasound evaluation.

**Fig. 9:**
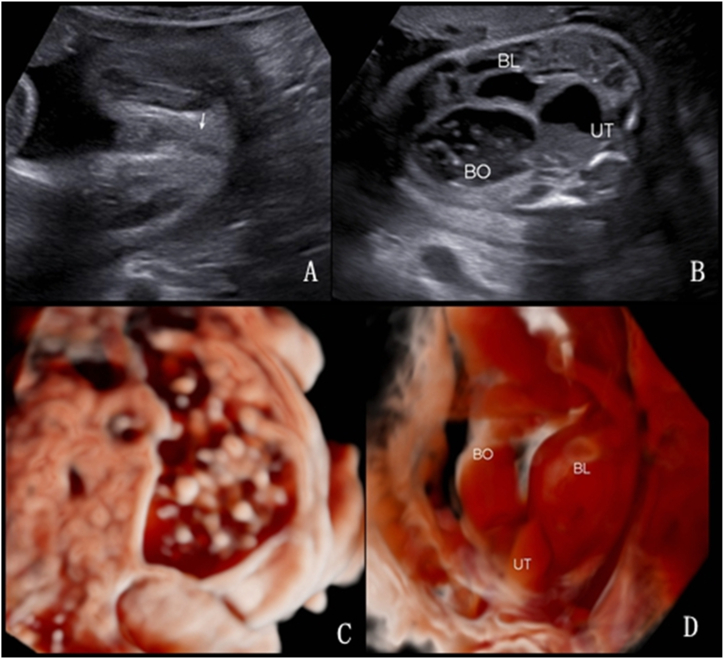
Urorectal septum sequence at 31 weeks and 6 days of gestation: A. 2D ultrasound shows absence of the “target sign” in the anal area; B. 2D ultrasound shows strong echo spots within the bowel, uterine distension with fluid accumulation, and local deposition of small echogenic spots; C. 3D imaging in grayscale mode demonstrates strong echogenic spots suspended within the dilated bowel; D. 3D imaging in inversion mode shows the spatial relationship between the bladder, uterus, and dilated colon, with the uterus appearing as a unicompartimental shape.

**Fig. 10:**
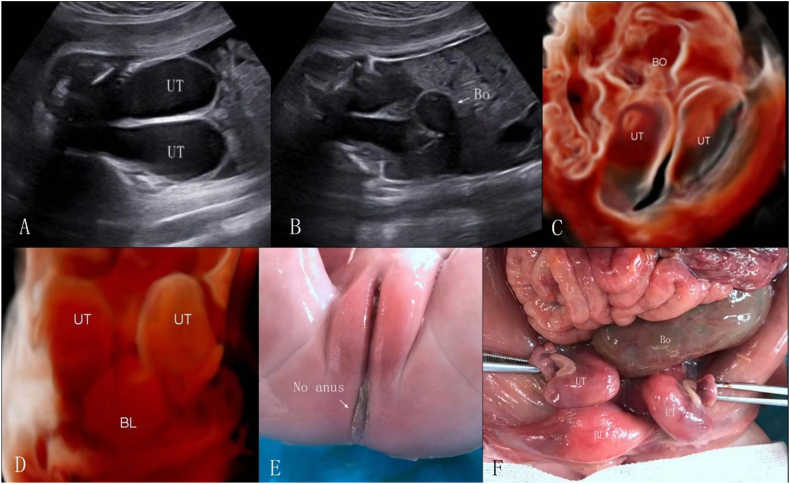
Urorectal septum sequence at 33 weeks and 3 days of gestation: A. 2D ultrasound shows the dilated uterus didelphys with effusion in the lower abdominal. B. 2D ultrasound shows colonic dilatation. C. In 3D Crystal Vue imaging grayscale mode, after increasing transparency, it displays the dilated colon located behind the uterus didelphys. D. In 3D inversion mode, it shows the double uterus located behind the distended bladder. E–F: After induction, anal atresia, left descending colon dilatation, and uterus didelphys effusion were confirmed.

Three cases of complex colonic dilatation were identified, and images of 2 of these are depicted in Fig. 9, Fig. 10. These cases were characterized by a urorectal septal malformation sequence combined with anal atresia, uterine malformations, uterine and vaginal dilatation with fluid accumulation, and ascites. Fig. 9 presents a urorectal septum sequence at 31 weeks and 6 days of gestation, showcasing the absence of the “target sign” in the anal area (Fig. 9A), strong echo spots within the bowel (Fig. 9B and C), and the spatial relationships among the bladder, uterus, and dilated colon (Fig. 9D). Fig. 10 presents a similar sequence at 33 weeks and 3 days of gestation, highlighting the dilated uterus didelphys with effusion (Fig. 10A), colonic dilatation (Fig. 10B), and the detailed anatomical relationships clarified by 3D imaging (Fig. 10C and D). The diagnoses of anal atresia, left descending colon dilatation, and uterus didelphys effusion were all confirmed post-induction (Fig. 10E and F).

**Fig. 11:**
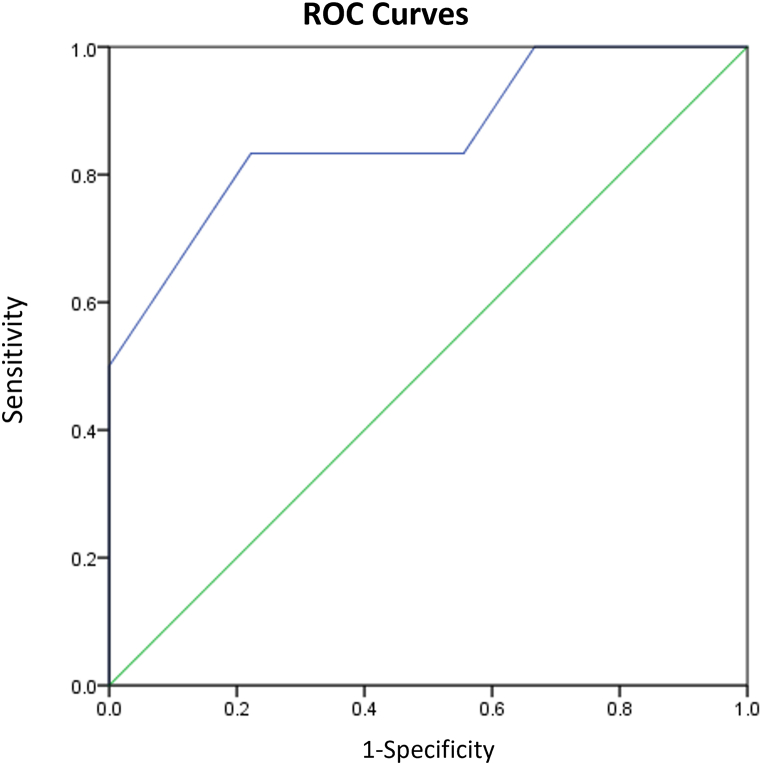
ROC curve of dilated colon diameter in predicting adverse pregnancy outcome in fetuses with colonic dilatation.

Postnatally, 9 cases exhibited normal development, while 2 underwent surgical treatment for proximal atresia of the ascending colon, and a diagnosis was confirmed in another 4 cases following labor induction. To predict adverse neonatal outcomes, colon diameter was used to generate a ROC curve (Fig. 11), which yielded an AUC of 0.861 (95% CI 0.655–1.0; p < 0.05). With an optimal cut-off of 21.5 mm, the sensitivity and specificity were 83.3% and 77.8%, respectively.

### 3.5 Evaluating obstruction at different locations using 2D and 3D ultrasound

Among the 68 cases with confirmed obstruction determined through surgery or labor induction, the ultrasound images and results were analyzed (Table 6). For duodenal obstructions, there was no difference in detection rates between 2D and 3D ultrasonography. However, 3D ultrasound demonstrated a significantly higher detection rate for jejunum/ileum (100% vs. 66.7%; p = 0.002) and colonic obstructions (100% vs. 50%; p = 0.045) than 2D ultrasound.

**Table 6:** Comparison between 2D and 3D ultrasound examination methods in identifying obstruction.

	Duodenal obstruction (n = 35)	Jejunum and ileum obstruction (n = 27)	Colonic obstruction (n = 6)
2D Ultrasound	35 (100%)	18 (66.7%)	3 (50%)
3D Ultrasound	35 (100%)	27 (100%)	6 (100%)
P value	1	0.002	0.045

### 3.6 Prediction model for postnatal surgical treatment

Among patients with duodenal dilatation, only 1 self-recovered, while the rest of the newborns underwent surgical treatment after birth.

**Fig. 12:**
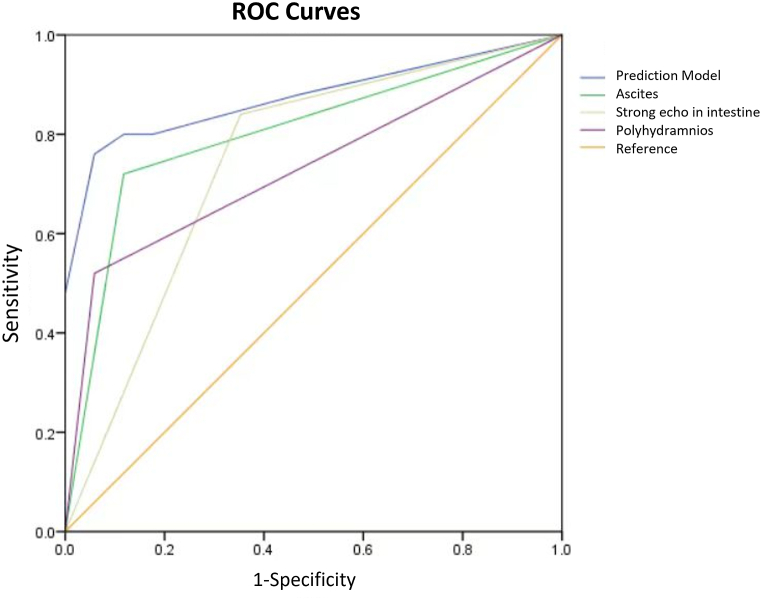
ROC curve of three-factor model in predicting neonatal operation in patients with fetal bowel dilatation.

For jejunum/ileum and colonic dilatation, using the backward stepwise selection method, a prediction model for neonatal operation in patients with fetal bowel dilatation was established: logit (P) = −1.58+(2.32 × polyhydramnios) +(2.0 × ascites) +(1.14 × hyperechogenic bowel). The AUC for the prediction model was 0.874 (p < 0.05), with 76% sensitivity and 94.1% specificity (Fig. 12). By inputting the values of these factors into the formula, healthcare professionals can estimate the probability (P) of neonatal surgery. There was a significant association between ascites and surgical treatments (odds ratio 7.449 [95% CI 1.03–53.7]; p < 0.05).

## 4 Discussion

The prenatal diagnosis of FBD is challenging. Using 2D ultrasound to differentiate between small intestinal and colonic dilatation remains difficult, as does determining the precise location of the obstruction [5]. Because dilatation at different locations can be associated with varying outcomes, providing an accurate prenatal diagnosis is crucial for appropriate management and treatment plans. In our cohort, the prevalence of FBD was 20 per 10,000 births. The dilatation rate was 8.3 per 10,000 for the duodenum, 8 per 10,000 for the jejunum and ileum, and 3.4 per 10,000 for the colon. The distribution of these three groups is consistent with previous research findings [[10], [11], [12], [13]]. However, our study found a higher overall prevalence, which may be attributed to the tertiary hospital's handling of complex cases and the use of advanced technologies for enhanced detection.

### 4.1 Prenatal diagnosis of pathological FBD

The double bubble sign has long been established as a valuable diagnostic tool for detecting fetal duodenal obstruction and is widely used in clinical practice [6,14]. Within our cohort, there was 1 false-positive case, whereas the remaining 35 exhibiting the double bubble sign were all confirmed as duodenal obstruction through surgical intervention or induced labor. This finding suggests the reliability of the double bubble sign as an ultrasound indicator of duodenal obstruction. To further prevent misdiagnosis, we recommend the dynamic application and monitoring of 2D ultrasound to observe the communication between the dilated intestinal tube and gastric bubble through the pylorus via lateral probe movement. Additionally, we used the 3D imaging inversion mode to examine the morphology of the dilated intestine and its connection to the pylorus. Lopze et al. [15] reported a case using a 3D imaging inversion mode to differentiate the gastric and duodenal cavities, demonstrated the communication between them (the pylorus), and suggested its potential in facilitating accurate volume assessment. In our study, we observed that the proximal duodenal obstruction exhibited a crescent moon shape change, while the distal obstruction exhibits a “C” shape change. These morphological distinctions can serve as additional tools for differentiating between duodenal and jejunal obstructions. Surgical diagnoses included duodenal diaphragm, annular pancreas, duodenal atresia or stenosis, and intestinal malrotation. This observation was consistent with findings from previous studies [16,17]. In our duodenal dilatation group, 94.7% (18 out of 19) experienced favorable postoperative outcomes, despite 68.4% undergoing preterm labor. It is closely related to an accurate and timely prenatal diagnosis.

To determine jejunum/ileum dilatation, we adopted the definition proposed by Nyberg et al. [7]: fluid-filled intestinal loops measuring at least 15 mm in length or 7 mm in diameter. However, the intestinal diameter of 8 patients with jejunum/ileum dilatation (23%) returned to normal during later follow-up. A similar trend was observed in the colonic dilatation group. Even when using 18 mm as the cut-off for diagnosing colonic dilatation, false-positive results still occurred, with 60% of cases resolving. This suggests that some dilatations are transient and represent physiological variations, rather than pathological abnormalities [8,18]. Therefore, we calculate the optimal cut-offs for pathological jejunum/ileum and colonic dilatation: 15.5 mm intestinal diameter for pathological jejunum/ileum dilatation (81.5% sensitivity and 62.5% specificity), and 21.5 mm diameter for pathological colonic dilatation (83.3% sensitivity and 77.8% specificity). We also generated a regression model for jejunum/ileum and colonic dilatation and found that polyhydramnios, ascites, and strong echoes in the intestine can be used to predict the need for neonatal operations, with 76% sensitivity, 94.1% specificity, and an AUC of 0.874. It is noteworthy that ascites was a significant predictor for neonatal operation. Persistent ascites increases the likelihood of neonatal surgical intervention. If ascites is gradually absorbed, it may indicate an improvement in the condition and a reduced risk for surgical intervention.

### 4.2 Characteristics and ultrasound signs of FBD

Compared with the other groups, duodenal dilatation was detected earlier in pregnancy, had the earliest delivery times, and resulted in the lowest birth weights. The higher the obstruction in the digestive tract, the earlier the symptoms tend to manifest. This difference in timing can facilitate an early prenatal diagnosis. The lower birth weights in this group are likely attributable to both the higher obstruction location, which limits nutrient absorption, and earlier delivery times. These findings are consistent with those of a previous study by Hall et al. [19] Another characteristic of FBD is that it can exist in isolation or coexist with other congenital abnormalities. In our study, the duodenal dilatation group exhibited the highest rate of chromosomal abnormalities, with trisomy 21 accounting for 77.8% of cases. Previous studies have indicated that trisomy 21 is found in one-third of newborns with duodenal atresia [20], and 3%–5% of patients with trisomy 21 present with duodenal atresia [21]. We also identified a patient with a combination of duodenal dilatation and *ADSL* mutations. This inherited metabolic disorder can lead to symptoms such as lack of spontaneous movement, seizures, and muscle weakness [22]. This syndrome is so rare that, to our knowledge, our report is the first to document a case combining an *ADSL* gene mutation with duodenal atresia. In addition, we found that approximately 30% of the patients with duodenal dilatation had abnormalities in other systems, with congenital heart anomalies the most common (45.4%). This aligns with the findings of Short et al. [23], who reported that 64.2% of the patients with congenital duodenal obstruction exhibited signs or symptoms suggestive of congenital heart disease. Other studies have also reported that more than one-half of fetuses with duodenal atresia are associated with other anomalies [21,24]. On the other hand, the jejunum/ileum dilatation group exhibited the lowest incidence of abnormalities in other systems, with no chromosomal abnormalities observed. This observation is consistent with a population-based public health report suggesting that jejunal/ileal atresia or stenosis is not typically associated with chromosomal abnormalities [25]. In the colonic dilatation group, 20% were complex cases, including 1 with VACTERL association and 2 with urorectal septum malformations. Previous research has indicated that bowel obstruction, particularly anorectal atresia, often presents with concurrent anomalies and is most commonly associated with VACTERL association or caudal regression syndrome [8,26]. When colonic dilatation is accompanied by other anomalies, bowel obstruction (particularly anal and rectal atresia) should be strongly suspected.

In detecting duodenal obstruction, there was no difference between 2D and 3D ultrasound because the double bubble sign on 2D ultrasound has promising diagnostic accuracy [6,14]. However, 3D volume imaging had a significantly better diagnostic efficiency than 2D ultrasound for recognizing and differentiating jejunum/ileum and colonic obstructions. 3D volume imaging has several valuable applications. Among the jejunal/ileal dilatation cases, approximately two-thirds were diagnosed with intestinal volvulus. Although the whirlpool sign is considered to be a typical ultrasound feature of intestinal volvulus in newborns and pediatric patients [27,28], prenatal diagnosis remains challenging in fetuses. We identified several ultrasound signs related to volvulus in our patients. First, the whirlpool sign, indicative of intestinal rotation around the mesentery [29], presented in 43.4% of fetuses with jejunal/ileal dilatation. The prevalence reported in the literature varies widely [30,31], likely due to the rarity of the disease and insufficient sample sizes. Not all patients presented with the classic pattern of the superior mesenteric vein around the artery, possibly due to inadequate perfusion leading to necrosis and intestinal perforation [32,33]. Furthermore, our observations suggest that the whirlpool sign can be transient, potentially changing, or disappearing as the disease progresses, indicating the potential for the spontaneous resolution of intestinal volvulus before birth. Second, the coffee bean sign was observed in one-third of the cases of jejunum/ileum dilatation. This is a classic sign of a closed-loop obstruction [34]. This sign can coexist with the whirlpool sign, or appear after the whirlpool sign disappears. Abnormal intestinal motility is also often observed. Intestinal necrosis was confirmed postnatally in all the patients exhibiting this finding. Third, the spiral sign was the most frequently observed among the 3 typical signs in volvulus fetuses, underscoring its importance as a key indicator for identifying this condition. In cases lacking typical signs of volvulus on 2D ultrasonography, the dilated intestines still present a spiral sign in the 3D inversion mode. 3D imaging in the inversion mode is not affected by intestinal necrosis and can effectively depict the shape of the dilated intestine when it is filled with fluid, whereas traditional 2D ultrasound may not provide detailed anatomical and pathological characteristics of fetal fluid-filled structures [35,36]. Fourth, the fluid-filled level sign was also observed in our cases. It is characterized by anechoic fluid above and echogenic material below (meconium and blood). The identification of a fluid-filled level within the dilated loops has also been shown to improve the accuracy of detecting suspected volvuli [30]. Fifth, necrotic changes in the intestinal wall often effaced the characteristic three-layer ultrasound pattern, instead presenting as a homogeneously hypoechoic single layer. The combined use of the Crystal Vue and Reality Vue techniques enables the depiction of the true volume of the anatomy and clarifies the structural boundaries.

In our complex colonic dilatation cases, 2 involved urorectal septum malformation sequences, characterized by the absence of perineal and anal openings, accompanied by ambiguous genitalia and urogenital, colonic, and lumbosacral anomalies [37]. Typical ultrasound signs include anal atresia, colonic dilatation (with or without enterolithiasis), fetal intra-abdominal cysts, and urinary tract anomalies, sometimes accompanied by ascites [38]. Given variations in the presentation of urorectal anomalies, prenatal diagnosis is challenging. We used 3D volume imaging techniques to invert the images of the dilated colon and cystic mass behind the bladder, enhancing the visualization of the uterine anomaly morphology and spatial relationships between the bladder, colon, and uterus. Adjusting the threshold and increasing tissue transparency in Crystal Vue imaging further clarified the organ adjacency. Additionally, in cases of enterolithiasis with uric acid stones, 3D Crystal Vue imaging in the grayscale mode can effectively depict the stones.

### 4.3 Differentiation between fetal small intestinal and colonic dilatation

While the double bubble sign demonstrates potential for detecting duodenal obstruction, its accuracy in diagnosing jejunum/ileum atresia is inconsistent, ranging from 25% to 63% [4]. Differentiating between small intestinal and colonic obstruction is also challenging. A previous study proposed using the echogenicity of intestinal fluid content as an indicator of the obstruction level in bowel atresia, linking lower obstruction levels with higher echogenicity [39]. However, our findings suggest that this assessment method does not consistently yield reliable results. Intestinal wall ischemia due to small intestinal obstruction often leads to reduced peristalsis. This can result in strangulated intestinal obstruction, in which the reciprocating motion of the contents in the small intestine causes increased echogenicity. Consequently, determining the location of the obstruction based solely on the echogenicity of bowel contents is ineffective. In our study, we propose the use of 3D volume imaging techniques to differentiate between small intestinal and colonic dilatation by accurately identifying the haustra of the colon. Traditional 2D ultrasonography lacks specific indicators for this purpose. Often, when the walls of two adjacent intestines come into contact and protrude into the intestinal lumen, they are mistakenly interpreted as the haustra of the colon on 2D ultrasound. However, 3D volume imaging provides a clearer visualization of the haustra of the colon (placing the sampling line on the inner wall of the dilated bowel). This method significantly improved the differentiation between small intestine and colonic dilatation. In our cohort, 3D volume imaging demonstrated a notably higher diagnostic efficiency than 2D ultrasound in distinguishing between jejunum/ileum and colonic obstruction, thereby enabling more accurate identification of the dilated small intestine and colon.

### 4.4 Limitations

This study had some limitations. First, because our center functions as a regional consultation hub for prenatal diagnosis, we may have observed a higher disease prevalence than previously reported, leading to potential selection bias and affecting the generalizability of our findings. Second, the constraints of being a single-center study with a relatively short duration and limited sample size restrict our capacity to delve into broader inquiries. Moreover, the design of this study focused on the measurements obtained at the initial diagnosis, without assessing the intra-individual variability in fetal bowel diameter(s). Future research necessitating longitudinal tracking of bowel diameter to comprehensively assess intra-individual variability and its implications for neonatal outcomes is required. The retrospective design of our study also meant that analyzing the variability in diagnostic accuracy among radiologists was beyond the scope of this investigation.

## 5 Conclusions

Duodenal dilatation occurs the earliest, with the highest incidence of chromosomal abnormalities and multi-system abnormalities. 3D ultrasound volume imaging has valuable applications in the diagnosis of bowel obstruction. Clinical signs, including polyhydramnios, ascites, and strong echoes in the intestine, can be used to predict neonatal surgery.

## Ethics statement

This study was reviewed and approved by the ethics committee of Anhui Province Maternity and Child Health Hospital, with the approval number: YYLL2021-03-01-02. Informed consent was obtained from all patients for participation in the study and for the publication of their anonymized case details and images. For participants under the age of consent (newborn infants), written informed consent was obtained from their parents or legal guardians.

## Funding statement

This research received supports from 10.13039/501100009558University Natural Science Research Project of Anhui Province of Anhui Provincial Department of Education, China (KJ2021A0351), and Health Research Program of Anhui Provincial Health Commission, China (AHWJ2023A30125).

## Data availability statement

Data will be made available on request.

## CRediT authorship contribution statement

**Xuelei Li:** Writing – original draft, Methodology, Funding acquisition, Formal analysis, Conceptualization. **Meng Zhou:** Visualization, Investigation, Data curation. **Shanshan Wang:** Software, Resources, Project administration. **Chaoxue Zhang:** Writing – review & editing, Validation, Supervision.

## Declaration of competing interest

The authors declare that they have no known competing financial interests or personal relationships that could have appeared to influence the work reported in this paper.
